# Supplementary material for: The genome sequence of a leaf beetle, Chrysomela saliceti (Weise, 1884) (Coleoptera: Chrysomelidae)
Source: Wellcome Open Res. 2025 Dec 22;10:688. [Version 1] doi: 10.12688/wellcomeopenres.25214.1 (PMC12881850; doi:10.12688/wellcomeopenres.25214.1)
Supplement: Peer review history

# Reviewer report: Reviewer response for version 1

**DOI:** 10.21956/wellcomeopenres.27790.r145670

**Published:** 2026-02-16

**Authors:** Somjit Homchan

**Affiliations:** 1 Naresuan University, Tha Pho, Thailand

Overall, this manuscript is well-written and presents a high-quality genome assembly. I have only a few suggestions and questions below.

 1) The background is too brief. Please provide more details on this species and its importance. Additionally, this section reads like an abstract that incorporates methods, leading to redundancy with later sections.

 2) The authors should address why this species' complete mitochondrial genome is unusually large compared to other beetles in the same family.

 3) Please explain while K-mer completeness of haplotype 1(82.53%) and haplotype 2(78.73%), when the combined both haplotypes it shows K-mer completeness  99.01%.

Are sufficient details of methods and materials provided to allow replication by others?

Yes

Is the rationale for creating the dataset(s) clearly described?

Yes

Are the datasets clearly presented in a useable and accessible format?

Yes

Are the protocols appropriate and is the work technically sound?

Yes

Reviewer Expertise:

Genetic markers, Population Genetics, Genomics

I confirm that I have read this submission and believe that I have an appropriate level of expertise to confirm that it is of an acceptable scientific standard.

# Reviewer report: Reviewer response for version 1

**DOI:** 10.21956/wellcomeopenres.27790.r145662

**Published:** 2026-02-09

**Authors:** Yu-Feng Huang

**Affiliations:** 1 Department of Computer Science and Engineering, Yuan Ze University (Ringgold ID: 34895), Zhongli District, Taoyuan City, Taiwan

This Data Note reports a high-quality chromosome-level assembly for a male *Chrysomela saliceti* generated using PacBio HiFi with Illumina Hi-C for scaffolding and phasing. The assembly is supported by strong algorithmic QC, including a k-mer–based QV (~60), high BUSCO completeness (99.0% for haplotype 1), Hi-C contact maps consistent with chromosome-scale scaffolds, and documented contamination screening and manual curation. Overall, the assembly meets (and exceeds) the stated Earth BioGenome Project benchmark summary (6.C.Q60).

 However, although the wet/dry workflows follow established DToL practices and the headline metrics are strong, several points require clarification to avoid misinterpretation - particularly regarding how phased haplotypes relate to completeness metrics and the structural validity of the mitochondrial genome. Specifically, the manuscript should (1) explain why haplotype-level k-mer completeness (~79–83%) is lower than the combined completeness (99%) in a phased assembly and clearly state which metric is used for EBP-style reporting; (2) briefly rationalize the small difference between the GenomeScope-estimated haploid genome size (~726 Mb) and the haplotype 1 assembly span (~739 Mb); and (3) explicitly confirm whether the 21.9 kb mitochondrial genome is circularized/complete and provide a brief explanation for its relatively large size. Adding a minimal annotation QC indicator and a short species-background paragraph would further strengthen the note.

 Major Comments

1. **Clarify k-mer completeness interpretation for phased assemblies**
  The manuscript reports haplotype-specific k-mer completeness of 82.53% (haplotype 1) and 78.73% (haplotype 2), while the combined haplotypes reach 99.01%. This pattern is generally expected for phased diploid assemblies (each haplotype captures only a subset of the total k-mer set), but it may confuse readers - especially since Table 4 juxtaposes “haplotype 1 summary” metrics with a combined-haplotype completeness benchmark. Please add a short explanation and explicitly indicate which completeness value is intended to support completeness claims and EBP-style reporting.
2. **Assembly span versus estimated genome size: briefly rationalize differences**
  GenomeScope2 estimates a haploid genome size of 726.46 Mb, whereas haplotype 1 spans 738.63 Mb. The ~12 Mb difference may be benign (e.g., unresolved repeats, small retained alternate segments, or minor residual duplication), but readers often interpret such discrepancies as evidence of duplication or incomplete haplotig purging. A brief statement clarifying plausible causes (and whether any residual duplication was assessed) would improve interpretability.
3. **Mitochondrial genome: confirm circularity/completeness and address unusually large size**
  The mitochondrial genome is reported as 21.9 kb, which is relatively large for many insects and raises the possibility of an expanded control region, duplicated segments, or a linear/incomplete assembly. Please explicitly state whether circularity was verified (e.g., by identifying overlapping ends and/or standardizing the circular start position). If available, a minimal validation (gene content presence or a brief note on the control region) would help alleviate concerns—particularly since the current text does not provide mitogenome annotation details.
4. **Gene annotation: add a minimal quality indicator beyond gene counts**
  The note reports 14,722 protein-coding genes, but does not provide a concise annotation QC indicator (even a single value). To strengthen claims of gene-level completeness/correctness, please include a minimal metric such as BUSCO on predicted proteins/transcripts or a brief statement about the evidence sources used in the annotation (e.g., homology and/or transcript support).

Are sufficient details of methods and materials provided to allow replication by others?

Yes

Is the rationale for creating the dataset(s) clearly described?

Yes

Are the datasets clearly presented in a useable and accessible format?

Yes

Are the protocols appropriate and is the work technically sound?

Partly

Reviewer Expertise:

Bioinformatics, Cancer Biology, Mitogenomics, Genome assembly and annotation

I confirm that I have read this submission and believe that I have an appropriate level of expertise to confirm that it is of an acceptable scientific standard, however I have significant reservations, as outlined above.

# Reviewer report: Reviewer response for version 1

**DOI:** 10.21956/wellcomeopenres.27790.r145663

**Published:** 2026-02-06

**Authors:** Wai Lok So

**Affiliations:** 1 The Chinese University of Hong Kong, Hong Kong, Hong Kong

This manuscript presents a high-quality genome assembly of *Chrysomela saliceti*. The sequencing and assembly methodologies are technically sound and logical. A well assembled haplotype 1, including the X sex chromosome was yield, together with a complete mitochondrial genome.The methods and processing pipelines are appropriate and transparent, which demonstrates replicability. The resources generated would be useful for research on Coleoptera and insect evolution, and related genomic studies. I would like to request for a scale bar on Figure 1, otherwise I have no major concerns of the manuscript.

Are sufficient details of methods and materials provided to allow replication by others?

Yes

Is the rationale for creating the dataset(s) clearly described?

Yes

Are the datasets clearly presented in a useable and accessible format?

Yes

Are the protocols appropriate and is the work technically sound?

Yes

Reviewer Expertise:

Evolution, Genomics, Insects, Myriapods

I confirm that I have read this submission and believe that I have an appropriate level of expertise to confirm that it is of an acceptable scientific standard.

# Reviewer report: Reviewer response for version 1

**DOI:** 10.21956/wellcomeopenres.27790.r144143

**Published:** 2026-01-27

**Authors:** Nikoletta Andrea Nagy

**Affiliations:** 1 University of Debrecen, Debrecen, Hungary

The manuscript is well written, and the genome presented is of high quality and can contribute to future research on leaf beetles. I have only a few suggestions for the authors.

 Background

 The background seems to be too short for me. I would recommend providing an additional paragraph which briefly describes the species.

 Figure 1

 Please put a scale on the photo

 Assembly statistics

 I would recommend including whether the assembled mitochondrial genome is an incomplete linear or a complete circular sequence.

Are sufficient details of methods and materials provided to allow replication by others?

Yes

Is the rationale for creating the dataset(s) clearly described?

Yes

Are the datasets clearly presented in a useable and accessible format?

Yes

Are the protocols appropriate and is the work technically sound?

Yes

Reviewer Expertise:

arthropod genomics, transcriptomics, behavioural genetics

I confirm that I have read this submission and believe that I have an appropriate level of expertise to confirm that it is of an acceptable scientific standard.
